# Advancing shipping NO*_x_* pollution estimation through a satellite-based approach

**DOI:** 10.1093/pnasnexus/pgad430

**Published:** 2023-12-11

**Authors:** Zhenyu Luo, Tingkun He, Wen Yi, Junchao Zhao, Zhining Zhang, Yongyue Wang, Huan Liu, Kebin He

**Affiliations:** State Key Joint Laboratory of Environment Simulation and Pollution Control, School of Environment, Tsinghua University, Beijing 100084, China; State Key Joint Laboratory of Environment Simulation and Pollution Control, School of Environment, Tsinghua University, Beijing 100084, China; State Key Joint Laboratory of Environment Simulation and Pollution Control, School of Environment, Tsinghua University, Beijing 100084, China; State Key Joint Laboratory of Environment Simulation and Pollution Control, School of Environment, Tsinghua University, Beijing 100084, China; State Key Joint Laboratory of Environment Simulation and Pollution Control, School of Environment, Tsinghua University, Beijing 100084, China; State Key Joint Laboratory of Environment Simulation and Pollution Control, School of Environment, Tsinghua University, Beijing 100084, China; State Key Joint Laboratory of Environment Simulation and Pollution Control, School of Environment, Tsinghua University, Beijing 100084, China; State Key Joint Laboratory of Environment Simulation and Pollution Control, School of Environment, Tsinghua University, Beijing 100084, China

**Keywords:** shipping emission, satellite observation, air quality impacts

## Abstract

Estimating shipping nitrogen oxides (NO*_x_*) emissions and their associated ambient NO_2_ impacts is a complex and time-consuming task. In this study, a satellite-based ship pollution estimation model (SAT-SHIP) is developed to estimate regional shipping NO*_x_* emissions and their contribution to ambient NO_2_ concentrations in China. Unlike the traditional bottom–up approach, SAT-SHIP employs satellite observations with varying wind patterns to improve the top–down emission inversion methods for individual sectors amidst irregular emission plume signals. Through SAT-SHIP, shipping NO*_x_* emissions for 17 ports in China are estimated. The results show that SAT-SHIP performed comparably with the bottom–up approach, with an *R*^2^ value of 0.8. Additionally, SAT-SHIP reveals that the shipping sector in port areas contributes ∼21 and 11% to NO_2_ concentrations in the Yangtze River Delta and Pearl River Delta areas of China, respectively, which is consistent with the results from chemical transportation model simulations. This approach has practical implications for policymakers seeking to identify pollution sources and develop effective strategies to mitigate air pollution.

**Significance Statement**

The top–down cognition of shipping nitrogen pollution is important to mitigate air pollution. In this study, we developed a novel model called SAT-SHIP to estimate regional shipping nitrogen oxides emissions and their contribution to ambient NO_2_ concentrations in China. Emissions estimated by SAT-SHIP performed comparably with the bottom–up approach, and the estimated air quality impacts are consistent with the results from chemical transportation model simulations. This simplified and efficient method will benefit a wide range of researchers and policymakers, aiding in the identification and mitigation of air pollution.

## Introduction

Coastal ports, as the “maritime gateway” to the countries, have forged a bond between the economy and culture, owing to their flourishing commerce, convenient transportation, and abundance of employment opportunities. However, the health of individuals congregating in port areas is also threatened by the severe air pollution (1–4). Despite considerable efforts made in recent decades to control air pollution, many harbor areas are still facing air pollution episodes with regular exceedances of World Health Organization guidelines or local regulations (5–7). In some developing countries in Asia, the atmospheric pollutant concentration in port areas can still exhibit higher values despite already high background concentrations (8, 9). This harbor air pollution such as sulfur dioxide (SO_2_) and nitrogen dioxide (NO_2_) should be allocated 30 to 50% culpabilities to shipping emissions (10, 11). As the mitigation of SO_2_ and particulate matter (PM) emissions from ships has been effectively achieved through the global sulfur cap and the implementation of regional emission control policies (12–14), shipping nitrogen oxides (NO*_x_*) emissions have emerged as the primary pollutant affecting port air quality and public health due to the lack of effective control and regulatory measures. In particular, the transition of ship fuel to natural gas as an alternative fuel for reducing carbon emissions may result in even higher NO*_x_* emissions (15, 16). Hence, it is imperative to prioritize the monitoring and reduction of shipping NO*_x_* emissions and their associated air quality impact in port areas.

It is a global technological challenge to evaluate and control NO*_x_* emissions from mobile sources in real-world operating conditions. For example, reducing NO*_x_* has proved more challenging for diesel vehicles; there is a growing gap between real-world NO*_x_* emissions and certification limits under the tightened emission limits (17). Similarly, the current approach for controlling NO*_x_* emissions from ships is still to certify emissions from new engines operating on engine test bench under the ISO 8178 D2 test cycle or ISO 8178 E3 test cycle (18). Nevertheless, there can be substantial differences between actual operating conditions and those used for certification. Unlike SO_2_ and PM emissions linked directly to fuel consumption, the production of thermal NO*_x_* is contingent upon combustion efficiency rather than fuel consumption. Consequently, the NO*_x_* emission factor may demonstrate considerable fluctuations due to the intricate operational modes of ships (19, 20). However, conducting large-scale on-board measurements is a formidable challenge. It is estimated that such measurements have been conducted on no more than 1,000 vessels worldwide, with the majority being small vessels and under fragmented operating conditions; thus, the representativeness of these measured data is limited. Consequently, the uncertainty in the NO*_x_* emission assessment continually accumulates during the bottom–up calculation, rendering the total emissions difficult to validate. Additionally, the bottom–up approach to estimate NO*_x_* emissions by combining activity data and emission factors is a complex and time-consuming task that often results in a significant time lag between the occurrence of emissions and the final completion of inventories. As a result, the current shipping NO*_x_* emissions remain uncertain and time lagging, impeding a comprehensive understanding of their impact on air quality.

Exploring alternative approaches is imperative for validating ship NO*_x_* emissions. It appears that satellites may be the optimal solution as they offer stable and nearly global coverage of atmospheric concentration observations (Fig. S1A). Therefore, the top–down inventory estimates driven by satellite observations may offer a new avenue to understand the total amount of shipping NO*_x_* emissions. At the early stage, the top–down approach is basically based on the chemistry transport model (CTM) by correcting prior emission inventories using the sensitivity of concentrations to emissions (21–23), which do not address the accumulated uncertainty in the bottom–up emission inventory calculations. Moreover, these studies generally focus on regional or global scales and introduce additional uncertainties in the simulation process (24). In 2011, the exponentially modified Gaussian (EMG) approach was introduced by Beirle et al. (25) as a pioneering technique to estimate NO*_x_* emissions from mixed emission sources in Riyadh, setting the stage for CTM-free approaches. Subsequently, this method has been widely adopted and stimulated the use of other CTM-free approaches, e.g. the mass balance method (26–28). This CTM-free approach is independent of the bottom–up estimates and has a lower computational cost and higher accessibility. In recent years, it has surpassed CTM-based methods in terms of development momentum (Fig. S1B). However, identifying ship emissions in port areas poses a greater challenge, as these locations are situated within heavily polluted atmospheric backgrounds that are influenced by human land–based emissions. The plumes from different emission sources are intermingled, making it difficult to discern the NO_2_ increment caused by ship emissions. Therefore, as of now, there are no CTM-free methods available that can successfully estimate shipping NO*_x_* emissions in port areas.

**Fig. 1.**
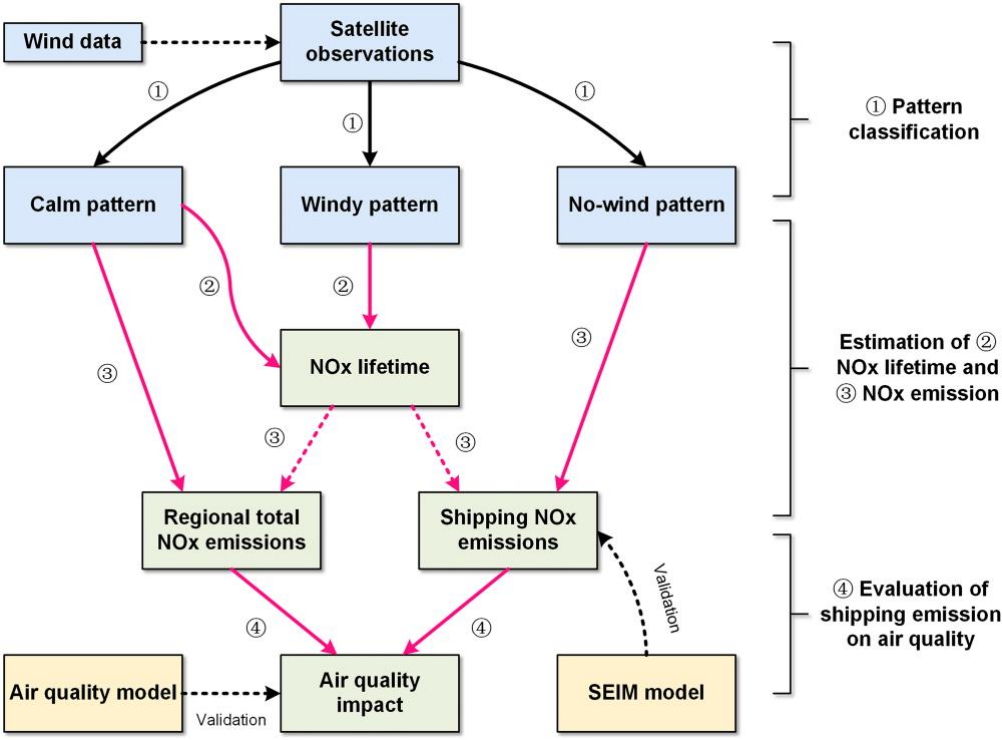
The framework of SAT-SHIP.

In this study, we develop a satellite-based ship pollution estimation model (SAT-SHIP) for estimating shipping NO*_x_* emissions and their associated NO_2_ concentrations by utilizing the varying distributions of satellite observations classified by wind patterns (Fig. 1). This approach offers several technical advancements, including (i) eliminating the uncertainties associated with bottom–up methods by not using emission inventories as a priori information; (ii) expanding the satellite identification range from areas with clearly distinguished hot spot signals, e.g. Riyadh (25), to areas with irregular signals and complex polluted atmospheric backgrounds; and (iii) advancing top–down inversion methods from estimating total emissions from mixed sources to individual sources. Based on the NO_2_ tropospheric vertical column densities (TVCDs) obtained from the Sentinel-5P/TROPOMI satellite for the year 2019, we estimate the shipping NO*_x_* emissions for 17 ports in China, including 4 of the top 10 ports in the world in terms of throughput (29), and further explore the impact of shipping emissions occurring in port areas on NO_2_ concentrations in coastal areas. We simultaneously use the advanced Ship Emission Inventory Model (SEIM), which utilizes the annual automatic identification system (AIS) (30) data of up to 30 billion records and static information for over 380,000 vessels worldwide, to provide shipping NO*_x_* emissions for the above ports. The performance of SAT-SHIP in estimating emissions and assessing air quality impact is validated through comparison with results from SEIM and the Weather Research and Forecasting Model with the Community Multiscale Air Quality (WRF-CMAQ) model. Throughout this study, we demonstrate that shipping NO*_x_* emissions can be directly resolved and quantified in polluted atmospheric environments using satellite observations. Although the current understandings of shipping NO*_x_* emissions from the bottom–up approaches are generally accurate in terms of magnitude, there might be a tendency toward an overly idealized representation when characterizing spatial distribution. Additionally, we suggest that the SAT-SHIP method can rapidly reflect the real-world impact of shipping emissions on regional NO_2_ pollution. This study has practical implications for policymakers seeking to identify pollution sources and develop effective strategies to mitigate air pollution.

## Results

We apply the SAT-SHIP model for determining port-level shipping NO*_x_* emissions and their impact on air quality. As illustrated in Fig. 1, the SAT-SHIP model involves several steps. First, the NO_2_ TVCDs are classified into different windy, calm, and no-wind patterns to investigate the distribution modes of plumes in port regions. Then, we estimate NO*_x_* lifetime and shipping NO*_x_* emissions for 17 ports in China using the line densities under different wind patterns, and this result is validated by SEIM. Finally, we estimate the total NO*_x_* emission and quantify the ship-related air quality impact using the derived NO*_x_* lifetime and line densities under the calm pattern, and this result is validated by WRF-CMAQ. The detailed methodology is presented in Materials and methods, and the results of each of the above steps are discussed below.

### Spatial distribution of NO_2_ TVCDs under different wind patterns

**Fig. 2.**
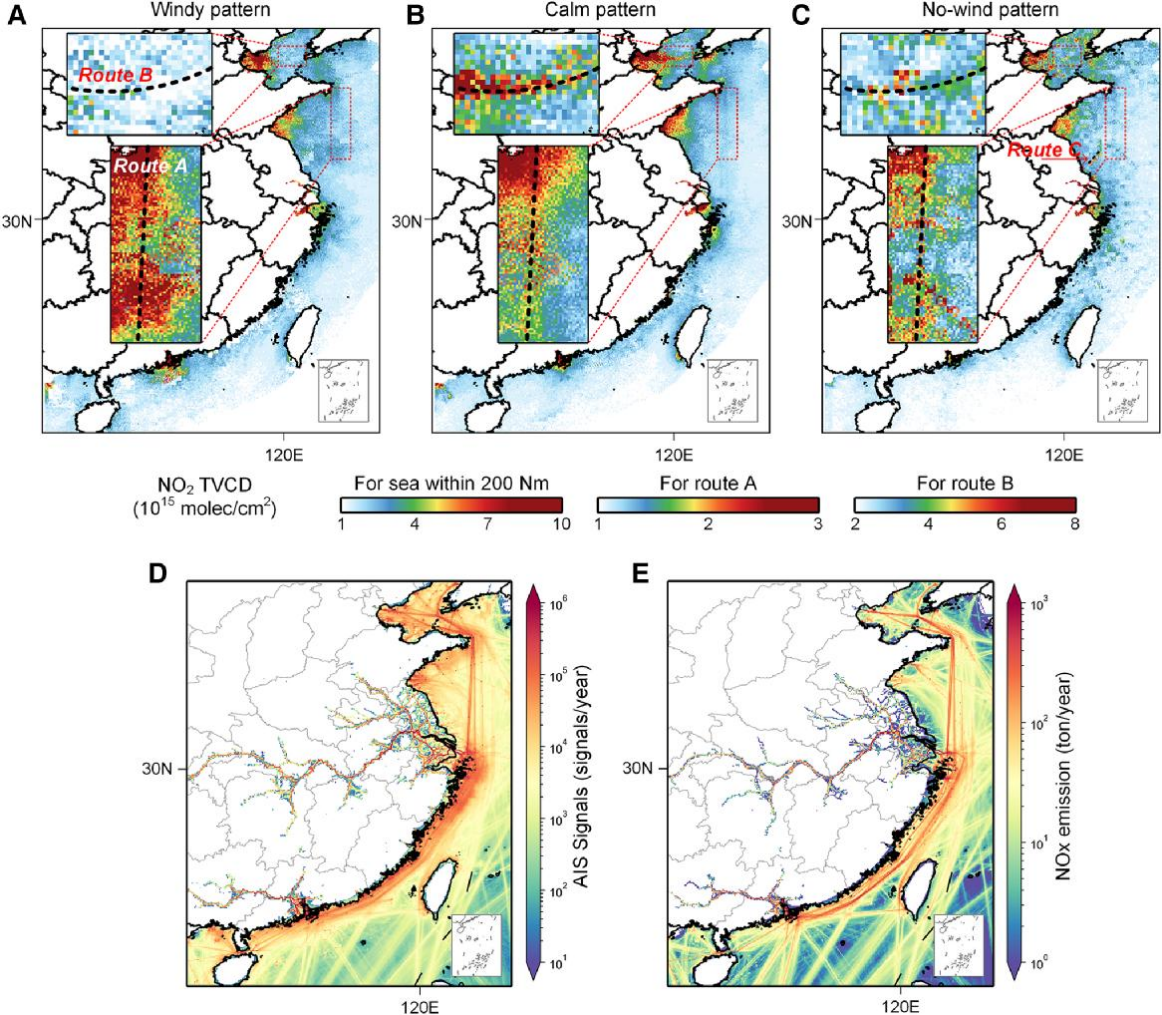
The spatial distribution of NO_2_ TVCDs under different wind patterns, activities, and emissions along the China coast for the year 2019. A) Northerly windy, B) northerly calm, and C) no-wind patterns. The D) AIS signals and E) shipping NO*_x_* emissions from SEIM for the year 2019.

Figure 2(A–C) illustrates the spatial distribution of NO_2_ TVCDs over the water surface within 200 nautical miles (Nm) from the Chinese mainland's territorial sea baseline under northerly windy, northerly calm, and no-wind patterns (definition in Materials and methods). In the windy pattern, it is shown that affected by the joint influence of land-based sources and the shipping sector, the high concentrations of NO_2_ over the sea surface are predominantly observed in port areas, such as the Bohai Rim Area (4), the Shandong Peninsula (SP), the Yangtze River Delta (YRD), and the Pearl River Delta (PRD). While in calm patterns, due to the reduced regional transportation from land areas, the signals over port area weaken, and the signals of some coastal or offshore routes such as route A and route B, as well as routes along southeast China, begin to emerge, which are basically indistinguishable in the windy pattern.

In the no-wind pattern, regional transportation is greatly limited, and the dispersion effect of NO_2_ TVCDs over port areas generally disappears. Instead, some strong signal points can be observed scattered throughout these areas. The outlines of shipping routes observed before in calm patterns become more apparent since the stronger signals can be observed in these routes compared with the background level, and the dispersion effect is further reduced. However, looking at each individual route, the signals appear chaotic and uneven in intensity, particularly in route A. This suggests that the real-world distributions of shipping NO*_x_* emissions along these routes depend on dynamic combustion efficiency, as opposed to the emissions provided by the AIS-based SEIM (Fig. 2D and E), which uses empirical adjustment factors to modify NO*_x_* emissions depending only on load factors, resulting in emissions that remain relatively constant with AIS signals along these routes (as shown in Fig. 2D). Additionally, a significant signal is detected along a new shipping route (route C) in the no-wind pattern (Fig. 2C), which is not captured by the SEIM. This may be attributed to the lack of signals for ships without deployed AIS equipment, such as small, old fishing vessels navigating in coastal waters. However, in port areas, satellite observations cannot detect the obvious route distribution or plume emissions from individual ships. This is due to the fact that ships in the port area are in different working modes and have no fixed routes. In conclusion, the findings suggest that the bottom–up approaches tend to estimate NO*_x_* emissions extremely empirically, using a constant emission factor according to fuel composition and engine type. However, the actual shipping emissions may vary significantly in intensity for each route, despite spatially basically following the distribution of AIS activity data.

### Port-level shipping NO*_x_* emission from SAT-SHIP

**Fig. 3.**
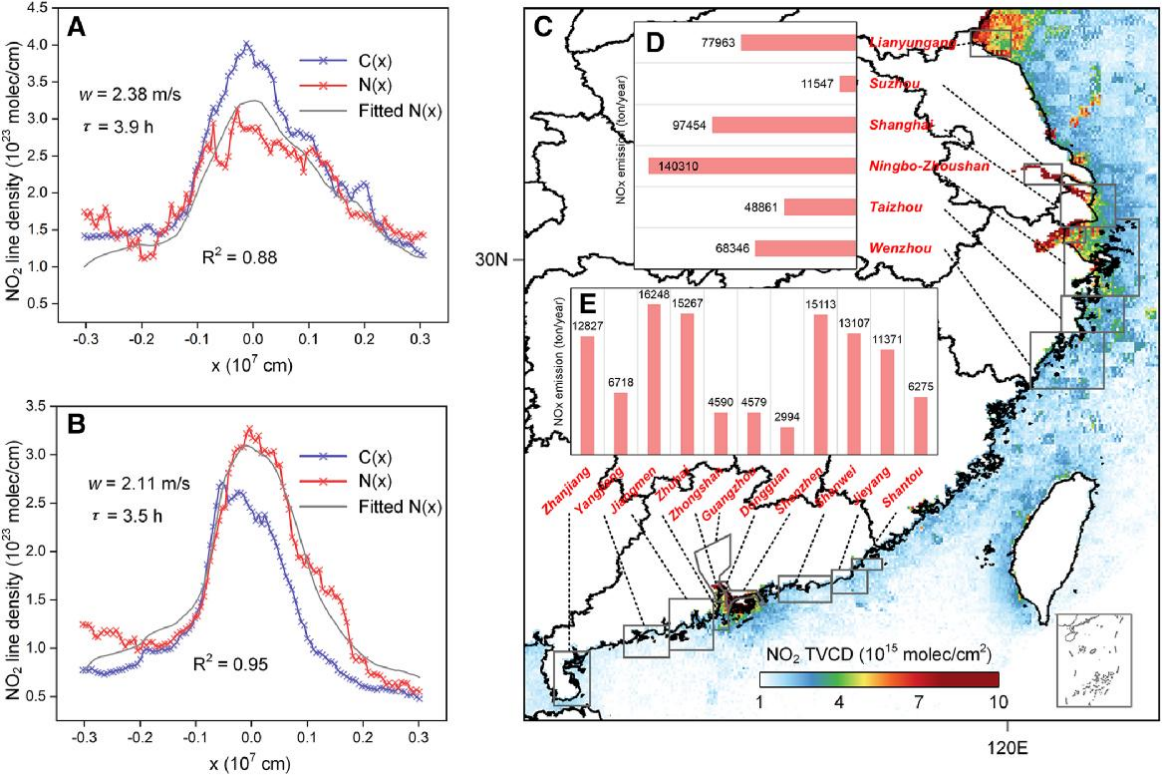
Estimation of lifetime and port-level emissions from SAT-SHIP. The NO_2_ line densities for windy pattern N(*x*) and calm pattern C(*x*) in A) YRD and B) PRD. The *w* represents the difference in wind velocities between windy and calm patterns, and the *τ* represents the derived NO*_x_* lifetime. C) The NO_2_ TVCDs no-wind pattern. The estimated shipping NO*_x_* emission for ports in the D) YRD region and E) PRD region. The gray boxes represent the boundary of those 17 ports in YRD and PRD, which are set according to the location of the ports and study by Wang et al. (13).

According to the mass balance concept, the total mass of NO*_x_* equals the emission rate times lifetime, and emissions can thus be derived in a three-step approach by (i) deriving the NO*_x_* lifetime over the port areas, (ii) calculating the total NO_2_ mass emitted from ships, and (iii) dividing the total NO_2_ mass by the lifetime. In this study, to estimate the atmospheric lifetime of NO*_x_*, we use the NO_2_ line densities in windy N(*x*) and calm patterns C(*x*) (the definition of line density is introduced in Materials and methods) and perform a nonlinear least-squares fit of N(*x*) using C(*x*) (described in Materials and methods). Figure 3(A and B) displays the observed line densities for windy and calm patterns around the YRD and PRD domains (Fig. S2A and B) and the fitted model function N(*x*). Generally, the model fit demonstrated good performance for both regions, with an *R*^2^ value of 0.88 and 0.95 for YRD and PRD, respectively. The resulting NO*_x_* lifetime in PRD is shorter than that in YRD, with values of 3.5 and 3.9 h, which are consistent with previous reports (Fig. S3). Furthermore, background NO_2_ TVCDs for YRD and PRD are estimated to be 1.5 × 10^23^ and 0.5 × 10^23^ molec/cm, respectively.

To calculate the total NO_2_ mass emitted from ships, the total NO_2_ TVCDs over the sea surface under no-wind patterns are utilized, and in combination with the derived lifetime, the shipping NO*_x_* emissions of major ports can be estimated (as described in Materials and methods). However, the NO_2_ TVCDs over ports located in BRA and SP are not involved since the NO_2_ TVCDs over these areas are hard to distinguish due to the large land-based emission from Beijing–Tianjin–Hebei region and Hebei Province, as well as the topography of enclosed inland seas. Additionally, ports in the YRD and PRD regions with annual NO*_x_* emissions <5,000 tons (as calculated in SEIM) are excluded as their emission intensities are too small to be detected by satellites. Ultimately, the emissions of 17 ports in the YRD and PRD regions (listed in Fig. 3C) are estimated (Fig. 3D and E). The estimated total shipping NO*_x_* emissions for the selected ports in the YRD and PRD regions are 444,481 and 109,091 tons/year, which represent ∼16 and 10% of the land-based anthropogenic emission in the YRD (including Shanghai, Jiangsu province, and Zhejiang province) and PRD regions from the MEIC model (http://www.meicmodel.org/ last access: March 2023). Ningbo-Zhoushan Port, with the world's highest throughput (29), is estimated to have an annual shipping NO*_x_* emission of 140,310 tons, which is significantly higher than emissions from other ports. Conversely, Dongguan Port has the lowest estimated emissions among these 17 ports.

### Comparison of port-level shipping NO*_x_* emissions from SAT-SHIP and bottom–up approach

**Fig. 4.**
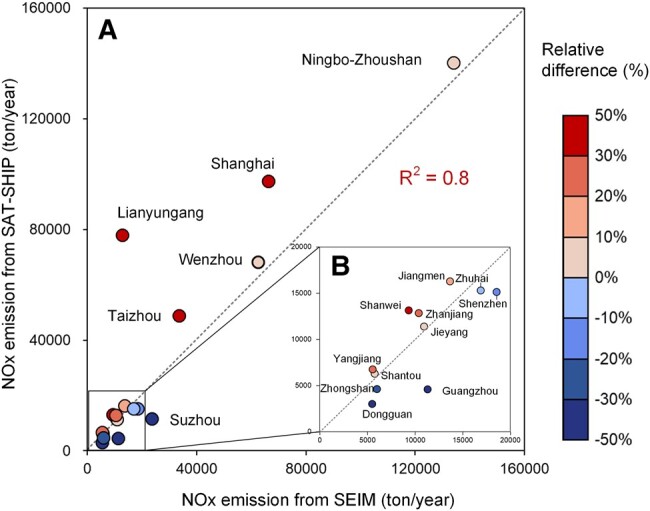
The comparison of results from SAT-SHIP and SEIM for A) the whole 17 ports and B) PRD. The color for points represents the relative difference in emissions from SAT-SHIP (Emis_SAT-SHIP_) and SEIM (Emis_SEIM_), which is calculated by (Emis_SAT-SHIP_−Emis_SEIM_)/Emis_SEIM_.

A comparison of the port-level NO*_x_* emission between SAT-SHIP and SEIM (described in Supplementary material) is conducted. In general, the coefficients of determination (*R*^2^) between emissions from SAT-SHIP and SEIM are 0.8 for these 17 ports in YRD and PRD, and all emissions from SAT-SHIP are within the ranges of ±50% around the SEIM, except for Lianyungang Port and Dongguan Port. This demonstrates the reliability of SAT-SHIP in capturing the total amount of shipping NO*_x_* emissions at the port level (Fig. 4). Specifically, the total emissions from SAT-SHIP are found to be 33% higher and 4.4% lower than those from SEIM for ports in YRD and PRD, respectively.

The discrepancies observed between SAT-SHIP and SEIM for each port are primarily attributed to the geographic location of these ports. Specifically, for ports with major waterways embedded in the land, including Suzhou Port, Dongguan Port, Zhongshan Port, Guangzhou Port, Zhuhai Port, and Shenzhen Port, the emissions estimated by SAT-SHIP are 38% lower than those obtained through SEIM. This is due to the exclusion of grids that cover over 80% of land areas in SAT-SHIP, which encompass most inland waterways and, consequently, fail to account for emissions emitted from ships operating in these areas. This limitation, however, is largely due to the underlying data, especially the spatial resolution of satellite observations, rather than the efficacy of inversion methods. For ports with distinct boundaries that are clearly delineated from the land, nearly all grids featuring ship activity can be included, and SAT-SHIP estimates a 37% higher emission than SEIM. This discrepancy can be attributed to three primary factors: (i) the emissions from ships without deployed AIS equipment are not included in SEIM; (ii) the NO_2_ arising from land-based sources is not completely excluded in the no-wind pattern utilized by SAT-SHIP; and (iii) SEIM does not consider the impact of oceanic conditions such as wind and wave on NO*_x_* emissions, which may induce speed loss and result in additional emissions (31). The effects of the former two factors should be insignificant, given that ships without AIS equipment are typically small vessels and emit lower amounts of NO*_x_*, and when using a threshold value of 0.2 m/s, the influence of land-based sources is mostly excluded (discussed in Materials and methods). And, the impact of oceanic conditions has not been explicitly quantified. As such, it is suggested that SAT-SHIP should perform better in estimating emissions for ports with clear boundaries to land. And for those ports with major waterways embedded in the land, SEIM is expected to yield more accurate results due to its reliance on AIS signals and lack of resolution concerns.

Moreover, it is important to note that the discrepancies between the two methods should be attributed to the uncertainties in both SAT-SHIP and SEIM. For the former, uncertainties have been discussed in Supplementary material. And, uncertainties in SEIM have been introduced in our previous work (13).

### Comparison of ship-induced NO_2_ concentrations from SAT-SHIP and WRF-CMAQ

**Fig. 5.**
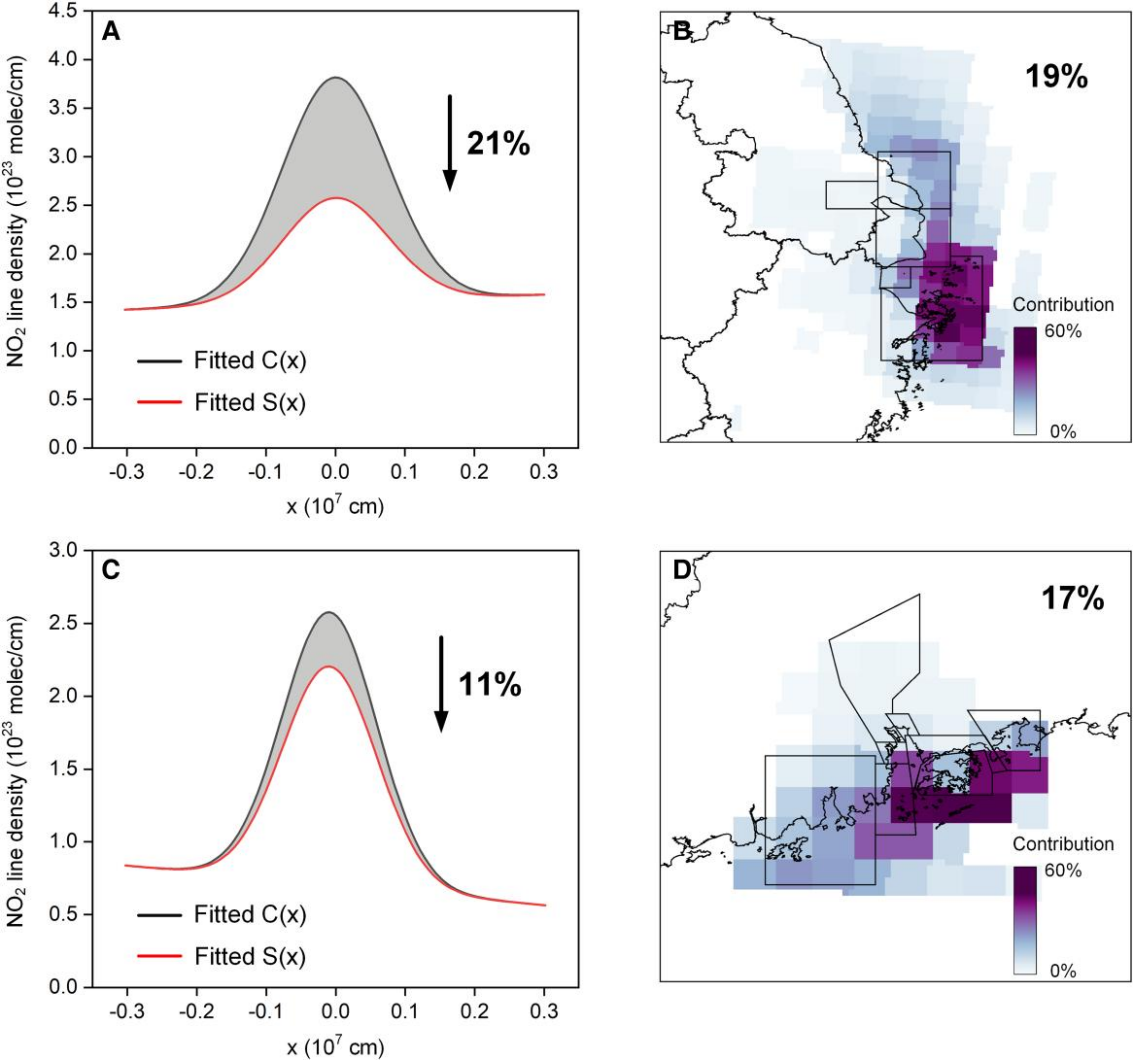
The impacts of shipping emissions on NO_2_ concentration for (A and B) YRD and (C and D) PRD from (A and C) SAT-SHIP and (B and D) WRF-CMAQ models (BASE-S1[2]/BASE). The black boxes represent the boundary of core ports of which the shipping emissions were excluded in the CMAQ simulation for scenarios S1 and S2 (introduced in Supplementary material). The fitted C(x) is the calm pattern line density plot using the fitted parameters in Fig. S7, and the S(x) is the line density plot using $A_{\mathrm{no}\text{-}\mathrm{ship},\;NO_{2}}$ and other fitted parameters in Fig. S7.

After comparing emissions, we attempt to approach the issue of the increment in NO_2_ concentration associated with shipping emissions occurring in port areas (black boxes shown in Fig. 5) from different perspectives. Employing a forward approach, we simulate the spatial distribution of ship-induced NO_2_ concentration using shipping emissions from SEIM via the WRF-CMAQ model (as delineated in Supplementary material). In addition, adopting an inverse approach SAT-SHIP, we evaluate the total ship-induced NO_2_ concentration at a regional scale by calculating the discrepancy between two Gaussian distributions based on the total NO_2_ mass and the total NO_2_ mass without shipping emissions estimated by SAT-SHIP (as described in Materials and methods).

Through the application of the SAT-SHIP, the examination of the impacts of port shipping emissions on NO_2_ concentration has revealed that such emissions result in a 21 and 11% increase in the YRD and PRD regions, respectively (Fig. 5A and C). This higher impact in the YRD region can be attributed to the higher proportion of shipping emission on total regional emission in YRD but also to the fact that the analysis is conducted in coastal land and water areas (red box in Fig. S2A), while the analysis conducted for the PRD region covers a larger area that extends northward to the northern Guangdong Province and southward to nearly 12 Nm (red box in Fig. S2B). This domain is the same as that for deriving lifetime; thus, when focusing on coastal areas, the effect of port shipping emissions on NO_2_ in the PRD areas should be more significant.

In terms of the average contribution calculated within the same domain as the SAT-SHIP (red boxes in Fig. S2), the WRF-CMAQ model simulates lower impacts of 19% for YRD and higher impacts of 17% for PRD, which can be mainly attributed to the differences in emissions from SAT-SHIP and SEIM, demonstrating that the SAT-SHIP could almost perform a comparable result with WRF-CMAQ, which considers physicochemical process when evaluating ship-induced NO_2_. In contrast, the WRF-CMAQ shows a more localized impact of shipping emissions on NO_2_ concentration. It shows that the impact of shipping emissions on NO_2_ pollution is primarily concentrated in regions over coastal waters with a contribution of over 40% (Fig. 5B and D). For inland areas near coastlines, this impact is about 10–15%, and it decreases as the distance from the coastlines increases.

It should be emphasized that currently, using SAT-SHIP is not possible to differentiate the spatial distribution of ship-induced concentrations based solely on the distribution of NO_2_ TVCDs in different patterns. Even in cases where there is no-wind pattern, the reduction of concentration on land grids not only involves the regional transportation effect of ship emissions but also includes the exclusion of emissions from other land-based grids. Therefore, the forward approach is used to validate the rationality of the SHIP-SAT, which is suggested to be used for regional-scale evaluation as it requires minimal computation and does not depend on prior emissions from various sources.

## Discussion

Accurately and timely understanding of shipping NO*_x_* emissions and their associated air quality is essential for the formulation of effective emission reduction and regulatory policies. In this study, we propose a satellite-based approach SAT-SHIP that advances top–down estimates from mixed sources to individual sources located in complex atmospheric environments without relying on prior information, to estimate shipping NO*_x_* pollution. By comparing the results from SAT-SHIP and SEIM, we first demonstrate that even in port areas with irregular plume tracks and complex polluted atmospheric environments, the NO*_x_* emissions from the shipping sector can be resolved and quantified using SAT-SHIP, and finding the current cognition on the amount of shipping NO*_x_* emission from bottom–up estimates is accurate in terms of magnitude. Additionally, in terms of the impact on air quality, we suggest that the SAT-SHIP can also accurately reflect the real-world impact of shipping emissions on NO_2_ concentration at a regional scale, which has been validated through CTM simulations.

As a top–down approach, SAT-SHIP operates independently of prior emission inventories. These inventories typically carry uncertainties stemming from various sources, including ship AIS data, emission factors, and empirical calculation formulas. Additionally, as a CTM-free inversion method, SAT-SHIP is free from the potential errors introduced by the modeled meteorological data, lifetimes, and emissions not only from the shipping sector but also from other anthropogenic and natural sources. While SAT-SHIP does entail some uncertainties of its own, the cumulative uncertainty is considerably lower than that of traditional approaches. This is primarily because SAT-SHIP involves fewer computational steps.

Moreover, SAT-SHIP significantly enhances the efficiency of computation. For example, SEIM requires several preprocessing steps such as data cleaning, route restoration, and missing value completion. Moreover, it necessitates emission calculation and results writing, which altogether takes around 5 days of computation for a global emission inventory at a resolution of 0.1° × 0.1°, using 24 cores on a computing platform with 128G computer memory. It is noteworthy that the time required for collecting underlying data such as ship activities and emission factors, which can take months or even years, is not involved in the above analysis. On the other hand, the WRF-CMAQ simulation takes around 8 days to run for the setup used in this study on the same computing platform as emission calculation. Consequently, when evaluating shipping emissions and their impact on air quality for global ports, using SEIM and WRF-CMAQ is extremely time-consuming. Comparably, SAT-SHIP can save over 99.9% of the calculation time and can be completed in several hours for this target.

It is also worth noting that all the raw data used in this study are publicly accessible, as detailed in the Data availability section. In contrast, the underlying data for traditional emission inventories are often nonpublic and challenging to obtain. The calculations in SAT-SHIP primarily involve raster image processing and fitting algorithms, which can be implemented using various geographic information systems and coding platforms, without the need for specialized modeling expertise. Consequently, this simplified and efficient approach offers advantages to a broader audience of researchers and policymakers, making it easier for them to engage with our findings.

There are also some unavoidable limitations in SAT-SHIP. As discussed before, SAT-SHIP cannot distinguish the shipping emissions from grids in which many waterways were embedded and thus would underestimate true emissions. Moreover, SAT-SHIP cannot reflect the spatiotemporal distribution of shipping emissions and air quality impacts. This is mainly due to the limitation in the resolution of satellite observations, not the method itself. At present, satellite spatial resolution is the biggest limitation of emission inversion methods. Therefore, developing approaches for superresolution (30, 32) in satellite observations or utilizing NO_2_ TVCDs with higher spatiotemporal resolutions is vital to address these problems. Additionally, enhancement in the time resolution of NO_2_ TVCDs may provide a new perspective in deriving shipping NO*_x_* emissions, such as implementing the estimation based on nonlinear chemistry occurring in ship plumes immediately after emission, which has been incorporated into CTMs such as GEOS-Chem (33). However, the development in time resolution is more complicated than spatial resolution.

Overall, we suggest the development of a comprehensive evaluation system that incorporates SAT-SHIP, SEIM, and air quality models. This system should aim to quantify the total emissions and characterize the heterogeneity of shipping pollution. Such an approach would offer benefits for research across multiple scales, from micro to macro, and hold significant potential for informing policy decisions (Fig. S4).

## Materials and methods

### Technical framework

The SAT-SHIP is a top–down approach using TROPOMI NO_2_ TVCDs and reanalysis wind data. It was developed to estimate shipping NO*_x_* emissions and their associated NO_2_ concentrations. The model involves several steps including pattern classification, lifetime time fitting, emission estimation, and air quality evaluation, which are illustrated in Fig. 1 and summarized below.

For NO*_x_* emissions estimation:

- The map of NO_2_ TVCDs is classified into windy pattern, calm pattern, and no-wind pattern according to the wind velocity of the corresponding grid.
- The atmospheric lifetime in coastal areas is derived based on NO_2_ TVCDs in the windy pattern and calm pattern using EMG model.
- The total mass of NO_2_ emitted from a ship is estimated based on NO_2_ TVCDs in the no-wind pattern, and then the emission rate is calculated based on the total mass of NO_2_ and lifetime derived in step (ii) using the mass balance approach.
- The emission calculated by our developed SEIM is used to validate the proposed model in this study.

For air quality assessment:

- The total NO*_x_* emission for the whole study area is derived using NO_2_ TVCDs in the calm pattern using the EMG model.
- The impact of shipping emissions on NO_2_ concentration in coastal areas is evaluated by calculating the difference in integration between the EMG function plotted by total NO*_x_* emission derived in step (v) and shipping NO*_x_* emission derived in step (iii).
- The results simulated by the CTM are used to validate the air quality impact derived in step (vi).

The specifics of the (i) pattern classification, (ii) fitting lifetimes, (iii) estimation of shipping NO*_x_* emission, and (v) and (vi) evaluation of shipping emission on air quality are introduced in the following sections, and the details of (iv) bottom–up ship emission modeling and (vii) WRF-CMAQ are introduced in Supplementary material.

### Pattern classification

In this study, NO_2_ TVCDs are classified into different patterns according to the wind velocity at pixel level. For example, the value of NO_2_ TVCD for a gird in the windy pattern is calculated by averaging values of this grid for all different days when the wind velocity meets the setting threshold. In this study, the threshold for different patterns is listed in Table S2. The threshold for distinguishing windy pattern and calm pattern is set following the previous studies conducted in China (34, 35). And, the setting of threshold for the no-wind pattern is referred as the value of dividing spatial resolution of NO_2_ TVCD by an approximate empirical annual average lifetime ($\frac{5.5\;\mathrm{km}}{4\;h}=0.38\;m/s$), where the polluted plume would not transport from the original grid to other grids within the lifetime, and the NO_2_ TVCDs over water grids basically come from shipping emissions and background concentration. Therefore, we conservatively set the threshold to 0.2 m/s in this study. Additionally, we evaluate the sensitivity of emissions for this threshold and will be detailed discussed below.

### Fitting lifetimes

The developed EMG model proposed by Liu et al. (34) is used in this study to derive the atmospheric lifetime in coastal areas. The NO_2_ TVCDs in calm and windy patterns represent the original distributions of NO*_x_* emissions and the transportive distributions of NO*_x_* emissions affected by wind, respectively. The lifetime can be derived by fitting the following function:


N(x)=a×[e⊗C](x)+b

where $e(x)=\exp\left(-\frac{x-X}{x_{0}}\right)$ is an exponential function for x≥0, otherwise is 0, $x_{0}$ is the *e*-folding distance downwind. *X* is the location of the source (relative to the a priori coordinates of the site under investigation). N(x) and C(x) represent the windy and calm line densities (molec/cm) calculated by the classified NO_2_ TVCDs patterns. Scaling factor *a* and offset *b* are used to account for possible systemic differences between calm and windy patterns.

The line density is calculated by integrating across the wind direction along the respective main wind direction to transform the 2D mean column density maps into 1D line densities, as illustrated in the study by Beirle et al. (25). As shown in Fig. S5, for each wind direction sector, the mean NO_2_ maps (orange, schematics) are integrated to fit range over the integration range, resulting in line densities (blue curve). In this study, the fit range represents the north–south direction interval of the red box depicted in Fig. S2, while the integration range represents the east–west direction interval of the red box shown in Fig. S2.

The wind speed threshold for filtering the no-wind pattern is stricter than that for the calm–wind pattern. As a result, many grid cells have significantly fewer NO_2_ column values filtered out in the no-wind pattern compared with the calm–wind pattern. Consequently, using the line density of no-wind pattern instead of the calm pattern to derive the lifetime may introduce more uncertainty due to insufficient data. Therefore, we have opted to use the “calm pattern” in our study to estimate the lifetime, ensuring a more robust analysis with a larger dataset and reduced data insufficiency uncertainties.

A nonlinear least-squares fit of N(*x*) is applied to derive *a*, *b*, and $x_{0}$. In this study, we set the fit interval in wind direction to 600 km and across-wind integration interval to 300 km, for the study domain (Fig. S2) in YRD (here the YRD region includes the partial areas of Shanghai, Jiangsu, and Zhejiang) and PRD, respectively. Next, the lifetime *τ* can be calculated by dividing $x_{0}$ by the average wind velocity *w*.

### Estimation of shipping NO*_x_* emission

In the EMG model, the emission source is generally regarded as a point source. Therefore, in coastal areas, the NO*_x_* emission derived from the EMG model will contain both land-based anthropogenic emissions and shipping emissions since all sectors in this area are considered as a big point source (discussed below). In this study, the mass balance approach is applied to estimate shipping NO*_x_* emission in the port, as follows:


$$E=\frac{A_{NO_{x}}}{\tau }=\frac{\sum_{S}\;\mathrm{TVC}D_{\mathrm{no}\text{-}\mathrm{wind}}-\mathrm{TVC}D_{\mathrm{BKG}}}{\tau }\times L$$

where $A_{NO_{x}}$ is the total NO*_x_* mass emitted from the ship, which is calculated by the difference between the NO_2_ TVCD at a no-wind pattern $\mathrm{TVC}D_{\mathrm{no}\text{-}\mathrm{wind}}$ and the background NO_2_ TVCD $\mathrm{TVC}D_{\mathrm{BKG}}$ over port areas *S*. *L* is the factor to scale NO_2_ to NO*_x_*. In this study, the grids with a proportion of land >80% are excluded. $\mathrm{TVC}D_{\mathrm{BKG}}$ is the intersection of N(x) and C(x) downwind in Fig. 2(A and B).

We calculated shipping NO*_x_* emission for each port using the proposed approach in this study with different thresholds of 0.5, 0.4, 0.3, and 0.2 m/s for the no-wind pattern. As shown in Table S1, the selection of threshold values does not result in significant fluctuations in the derived emissions. With a stricter threshold, the derived emissions tend to decrease, except for ports that are underestimated by the proposed model when compared with SEIM. This is because it becomes more challenging to differentiate land-based emissions (Fig. S6). The decline in emissions as the threshold tightens appears to occur at an inflection point between 0.3 and 0.4 m/s for most ports. Therefore, we believe that setting a threshold of 0.2 m/s is adequate for distinguishing ship emissions. Furthermore, it should be considered that a smaller threshold (<0.2 m/s) may lead to a pattern map with fewer missing values.

### Evaluation of shipping emission on air quality

According to Liu et al. (34), the NO*_x_* emission can be derived based on the calm line densities C(x), as follows:


$$C(x)=A_{NO_{2}}\times \frac{1}{\sqrt{2\pi }\sigma }\exp\;\left(-\frac{{\left(x-X\right)}^{2}}{2\sigma^{2}}\right)+\varepsilon +\beta x$$

where $A_{NO_{2}}$ is the total mass of NO_2_, *X* is the location of the source, $\sigma_{i}$ is the standard deviation, and ε+βx represents an offset and a possible linear gradient in the background field, respectively. The fitted $A_{NO_{2}}$ is scaled by 1.32 to obtain NO*_x_* mass since the typical concentration ratio of NO to NO_2_ is 0.32 in urban areas (36). Finally, the emission rate of NO*_x_* is calculated by dividing the total NO*_x_* mass by lifetime.

Considering that the small interval would not capture all plumes across wind direction due to dilution, the NO_2_ mass should be corrected by the following equation:


$$f(\sigma )=\frac{\int_{-300\mathrm{km}}^{300\mathrm{km}}\frac{1}{\sqrt{2\pi }\sigma }\exp\;\left(-\frac{{\left(x-X\right)}^{2}}{2\sigma^{2}}\right)}{\int_{-\infty }^{\infty }\frac{1}{\sqrt{2\pi }\sigma }\exp\;\left(-\frac{{\left(x-X\right)}^{2}}{2\sigma^{2}}\right)}$$

Hence, the top–down NO*_x_* emissions for YRD and PRD regions for the year 2019 are estimated and compared with the Multiresolution Emission Inventory for China (MEIC). As shown in Fig. S7, the correlation coefficients of the fit are 0.94 and 0.98 for YRD and PRD, indicating the reliability of our results. The emission rates are calculated at 396 and 319 mol/s for YRD (here is Shanghai, Jiangsu Province, and Zhejiang Province) and PRD, respectively, which is larger than those of in MEIC (35). This is because MEIC only includes the emissions from mobiles, industry, power, domestic, and agriculture, while the emissions from other sources such as shipping or open burning are not included. Therefore, the method based on the Gaussian function cannot be used for the estimation of NO*_x_* from a specific source.

Based on the method described above, we obtained the amount of NO_2_ mass ($A_{NO_{2}}$) and the fitted parameters. To investigate the impact of shipping emissions on air quality, we applied the concept of “zero-out” method, considering the impact of atmospheric chemical properties and background concentrations (37). Therefore, we further get the NO_2_ mass without port shipping emissions ($A_{\mathrm{no}\text{-}\mathrm{ship},\;NO_{2}}$) by subtracting the ship-induced NO_2_ mass from $A_{NO_{2}}$, which is calculated using SAT-SHIP. (In this study, the ship-induced NO_2_ mass is calculated in a range of black boxes in Fig. 5.) Then, we plot the S(x) using $A_{\mathrm{no}\text{-}\mathrm{ship},\;NO_{2}}$ and the fitted parameters in Fig. S7. By calculating the relative difference in integration between C(x) and S(x) (gray area in Fig. 5A and C), the contribution of shipping emissions to NO_2_ pollution can be quantified, which is further compared with the result simulated by CMAQ models. It is worth noting that the EMG model analyzes the contribution at a regional center level, treating all emissions as a single large point source. On the other hand, CMAQ provides contributions for each grid in the study domain.

## Supplementary Material

pgad430_Supplementary_Data

## Data Availability

Source data for wind in this paper are available in the ERA5 hourly data (https://cds.climate.copernicus.eu/, last access: August 2023), and source data for satellite observations are available in the TROPOMI level 2 offline NO_2_ data (https://sentinel.esa.int/web/sentinel/technical-guides/sentinel-5p/products-algorithms, last access: December 2023). All data for emission are included in the manuscript (Figs. 3 and 4) and/or Supplementary material (Table S1).
