# Supplementary material for: Advancing shipping NOx pollution estimation through a satellite-based approach
Source: PNAS Nexus. 2023 Dec 11;3(1):pgad430. doi: 10.1093/pnasnexus/pgad430 (PMC10745280; doi:10.1093/pnasnexus/pgad430)
Supplement: pgad430_Supplementary_Data [file pgad430_supplementary_data.docx]

**
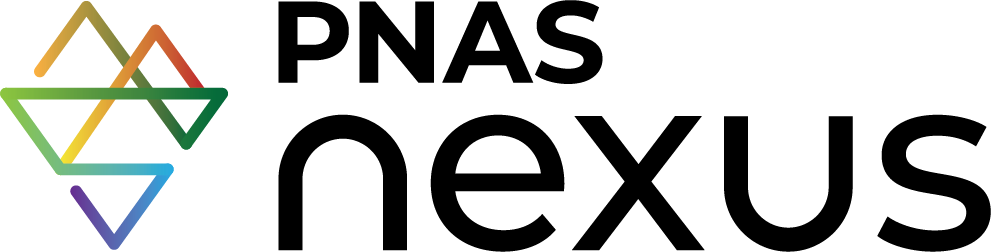
**

**Supplementary Information for**

Advancing shipping NOx pollution estimation through a satellite-based approach

Zhenyu Luo, Tingkun He, Wen Yi, Junchao Zhao, Zhining Zhang, Yongyue Wang, Huan Liu*, and Kebin He

*Huan Liu

**Email:**  liu_env@tsinghua.edu.cn

**This PDF file includes:**

Supplementary text

Figures S1 to S7

Tables S1 to S2

SI References

**Supplementary Information Text**

**Bottom-up shipping emission modelling**

The SEIM, which was established in our previous work, aims to develop a multiscale ship emission inventory with high spatial and temporal resolution by utilizing high-frequency automatic identification system (*1*) data. The emissions are calculated based on the instantaneous operating status and power changes for each individual ship between two successive AIS signals, usually lasting from a few seconds to a few minutes. Technical profiles for classification and emission calculation are dynamically matched with each active ship in AIS data. The total emissions are aggregated by that from all ships of all time intervals in the whole year, resulting in an inventory with high temporal and spatial resolution. The technical details such as data collection and cleaning, calculation formula, emission factor adoption, and default parameter setting is introduced in our previous studies (*2-4*). It considers ship emission for both air pollutants (e.g., SO_2_, PM, NO_X_, CO and HC) and greenhouse gases (e.g., CO_2_, CH_4_ and N_2_O) from main engines, auxiliary engines, and boilers.

SEIM is driven by driven (a) the high-frequency ship AIS data, including signal time, coordinate location, navigational speed, and operating status, and (b) the integrated Ship Technical Specifications Database (STSD) (updated to 2020), which describes ship static properties, including vessel type, maximum designed speed, DWT and engine power.

**Air quality modelling**

The Weather Research and Forecasting (WRF, version 3.8.1) − Community Multiscale Air Quality (CMAQ, version 5.3) model is applied to simulate the air quality in China during January, April, July and November for year of 2018, which represent winter, spring, summer and fall, respectively, with 3 days of spin-up time for each run. The modeling domain covered all of China and some parts of East Asia with a horizontal resolution of 36 km × 36 km, including all highly developed city clusters of China. The detailed configuration and validation of WRF and CMAQ is introduced in our previous work (*5*).

In order to evaluate the effects of shipping emissions on NO_2_ pollution, we conduct three distinct simulations in this research: (1) the inclusion of all emissions (BASE), (2) the exclusion of shipping emissions in core ports in the YRD region (S1), and (3) the exclusion of shipping emissions in core ports in the PRD region (S2), as indicated by black boxes in Fig. 4.

**Satellite observations and wind data**

The TROPOMI is a pushbroom spectrometer with spectral bands in the ultraviolet, visible, near infrared and shortwave infrared (*6*). It provides daily global coverage NO_2_ TVCDS of 3.5 km by 5.5 km (3.5 km by 7 km before August 2019) with overpasses near 13:30 local solar time. We used TROPOMI Level 2 offline NO_2_ data for 2019 (*7*). Daily maps are generated by aggregating the TROPOMI measurements to the original spatial resolution and filtering out pixels with quality assurance values < 0.5 and cloud fractions > 40%.

ERA5 is the fifth-generation European Center for Medium range Weather Forecasts (ECMWF) reanalysis for the global climate and weather for the past 4 to 7 decades. It provides global hourly estimates for a large number of atmospheric, ocean-wave and land-surface quantities at a 0.25° spatial resolution and 1 hour temporal resolution. Here the average wind vectors $w=\left( u,v \right)$ at a height of 10 m above the surface for the local time 13:00 and 14:00 are used.

**Uncertainty**

In this section, we will delve into the various sources of errors that contribute to the overall uncertainties in our results. (a) The uncertainty in tropospheric NO_2_ columns derived from satellite measurements is estimated to be around 30% (*7*), which is mainly attributed to uncertainties in the a-priori data, such as the relative vertical NO_2_ profiles, surface albedo, and cloud fraction. (b) The uncertainty in the lifetime fit due to uncertainties in both the speed and direction of the winds is estimated to be about 30% in Liu’s study (*8*). (c) For the lifetime fit, variations of the fit interval f or integration interval by 100 km result in lifetimes varying by only about 10% (*9*). (d) To obtain the total NOx emissions, the observed NO_2_ tropospheric columns need to be scaled up. We use a mean NOx/NO_2_ correction factor of 1.32, which comes with an estimated uncertainty of 10%. Because of the high solar irradiation and rapid vertical mixing, the NOx system reaches a steady state quickly, and any systematic downwind changes become insignificant (*10, 11*). (e) For estimation of shipping NOx emission by mass balance approach, the main source of uncertainty lies in the choice of threshold for identifying no-wind patterns, which is estimated to be 5%.


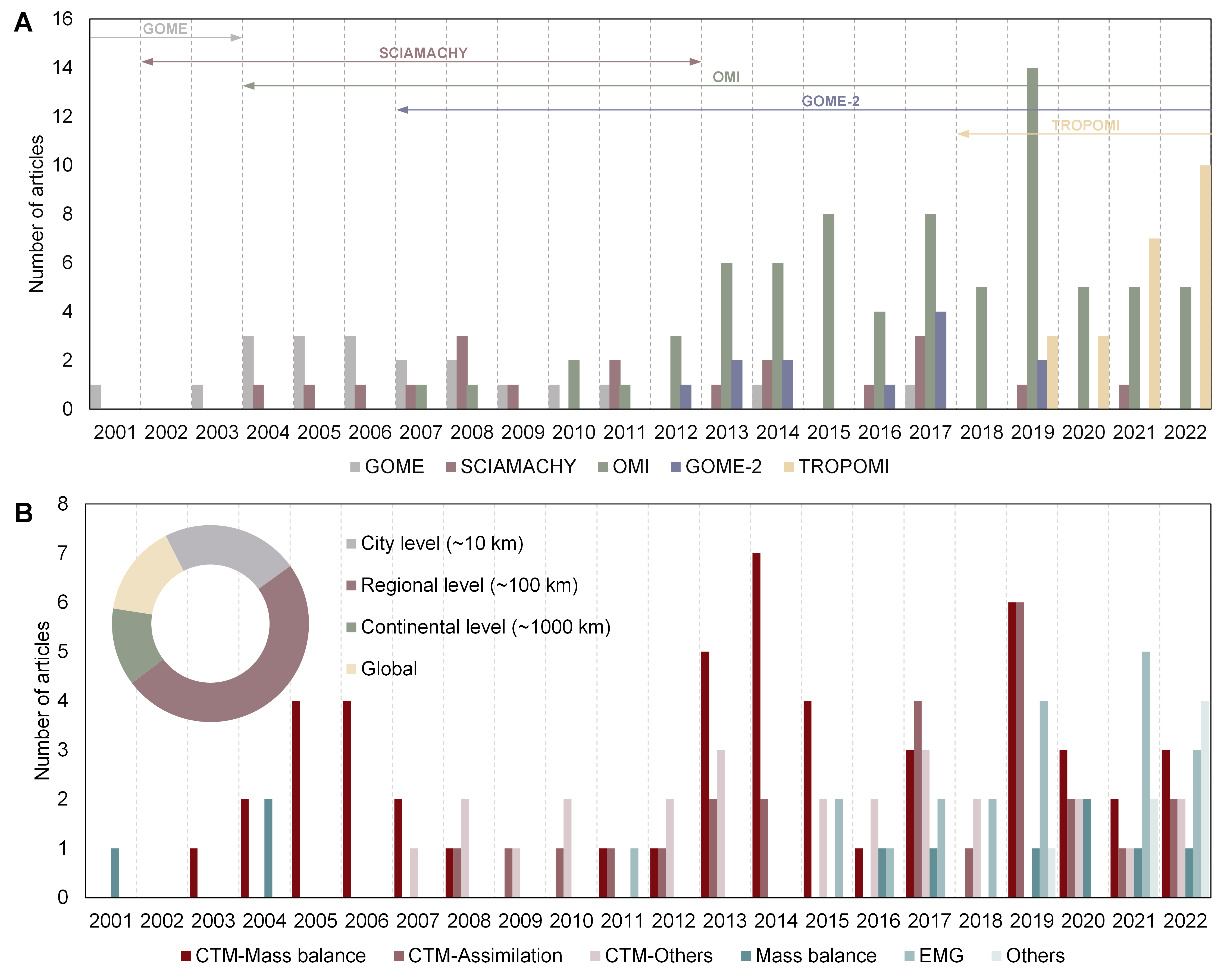


Fig. S1. Statistics of articles related to estimating NOx emissions through satellite observations. (A) The interannual variation of the number of studies with the development of satellite technology. (B) Methods and scales of these studies. Articles were retrieved through a systematic keyword search in Web of Science. Keyword searches included words pertaining to “NOx emission” and “Satellite”. The search included articles published from January 2001 to July 2022.

*Increasing application of the CTM-free method, especially the mass balance approach, can be seen from above.


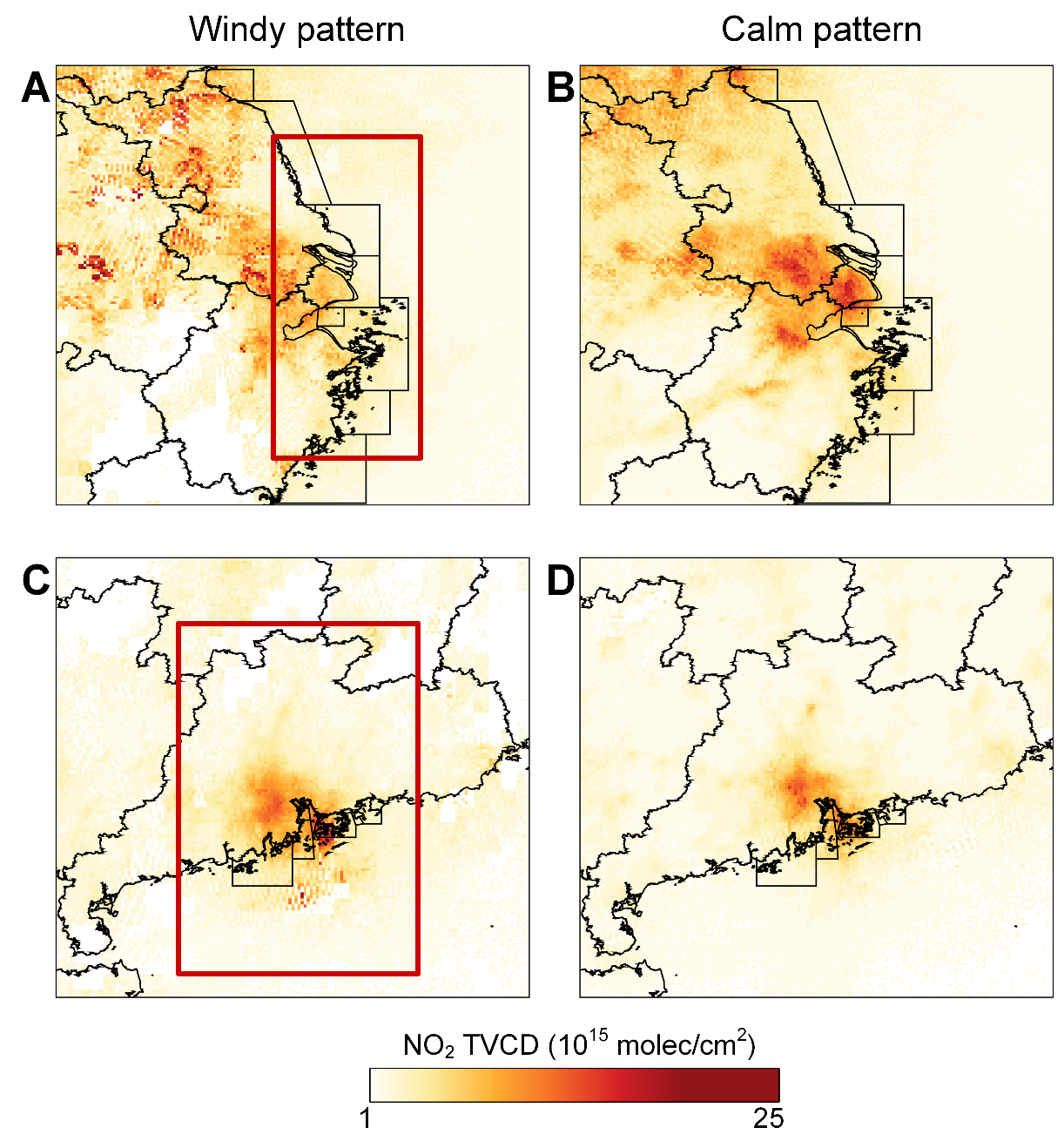


Fig. S2. The NO_2_ TVCDs pattern. (A, C) Northerly windy pattern, (B, D) calm patter for (A, B) YRD regions and (C, D) PRD regions. The red box represents the fit interval (north-south direction) and integration interval (east-west direction). The black boxes represent the boundary of core ports in these regions.


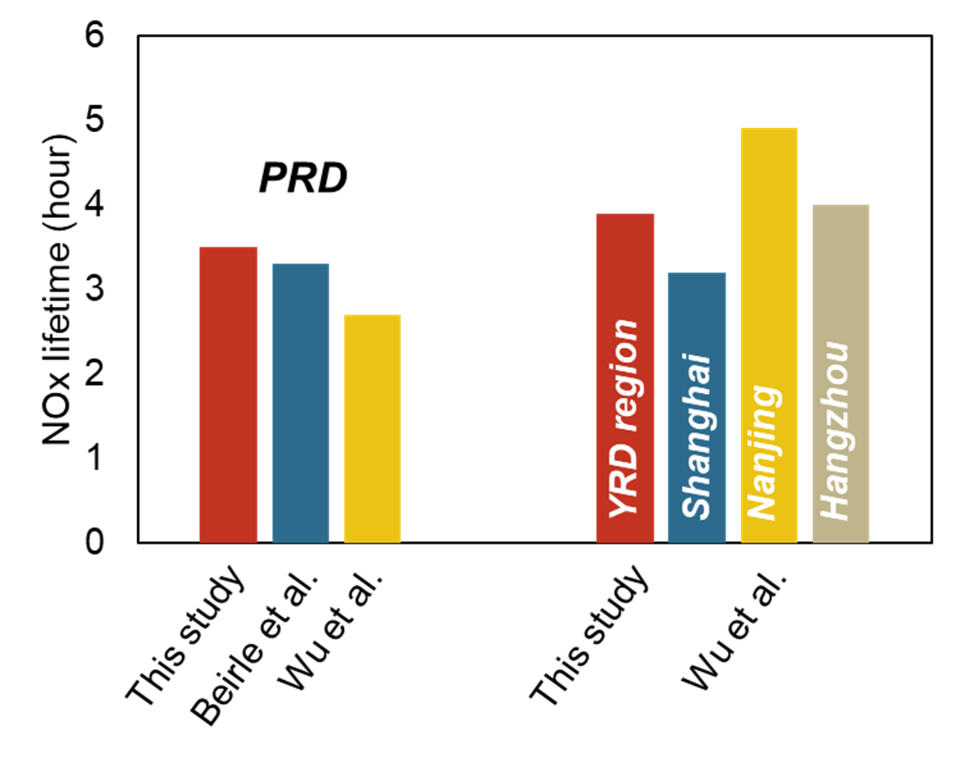


Fig. S3. Comparison of the derived lifetimes with other studies (*9, 10*).

**
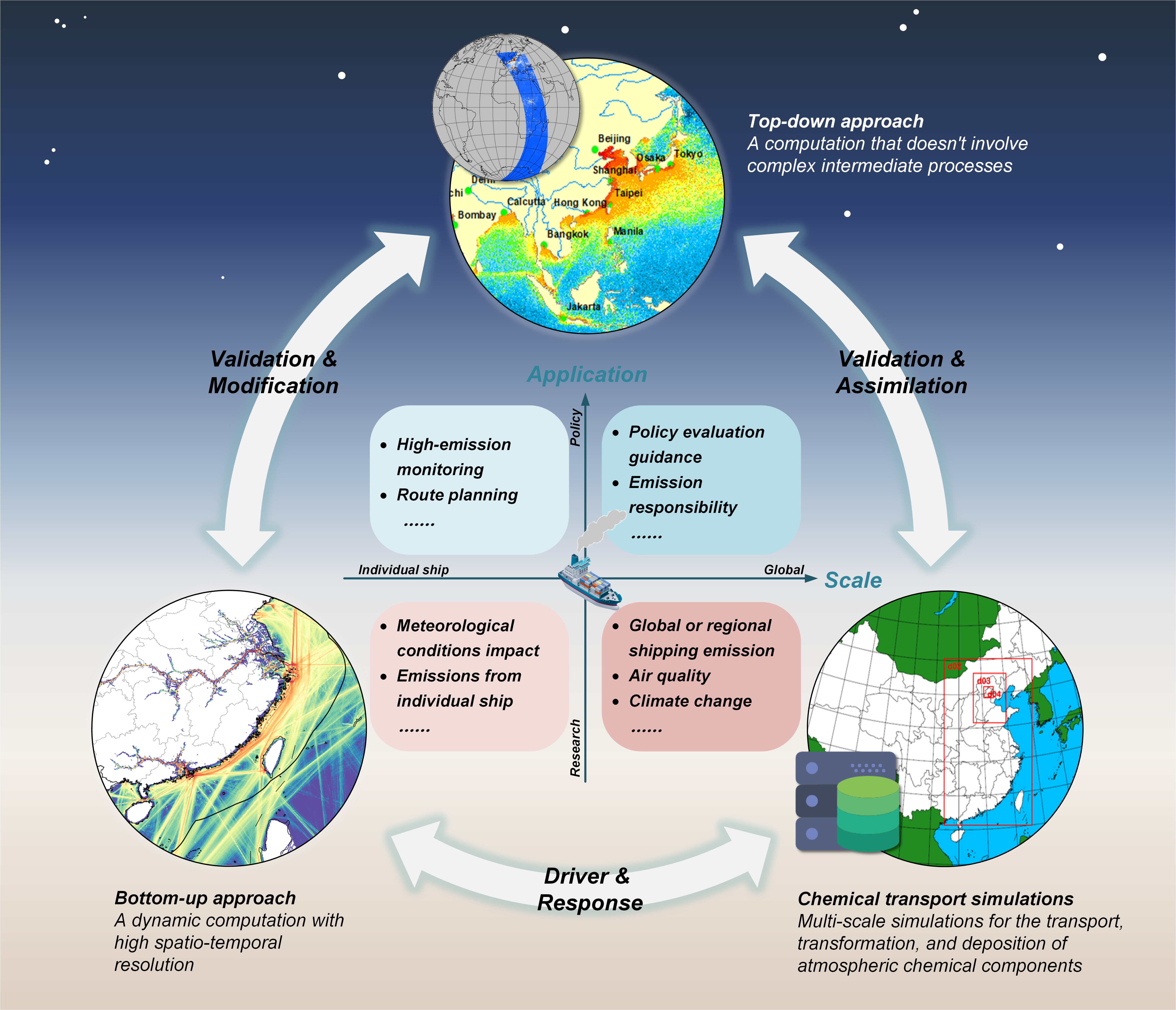
**

Fig. S4. A framework of ship pollution system and its application.


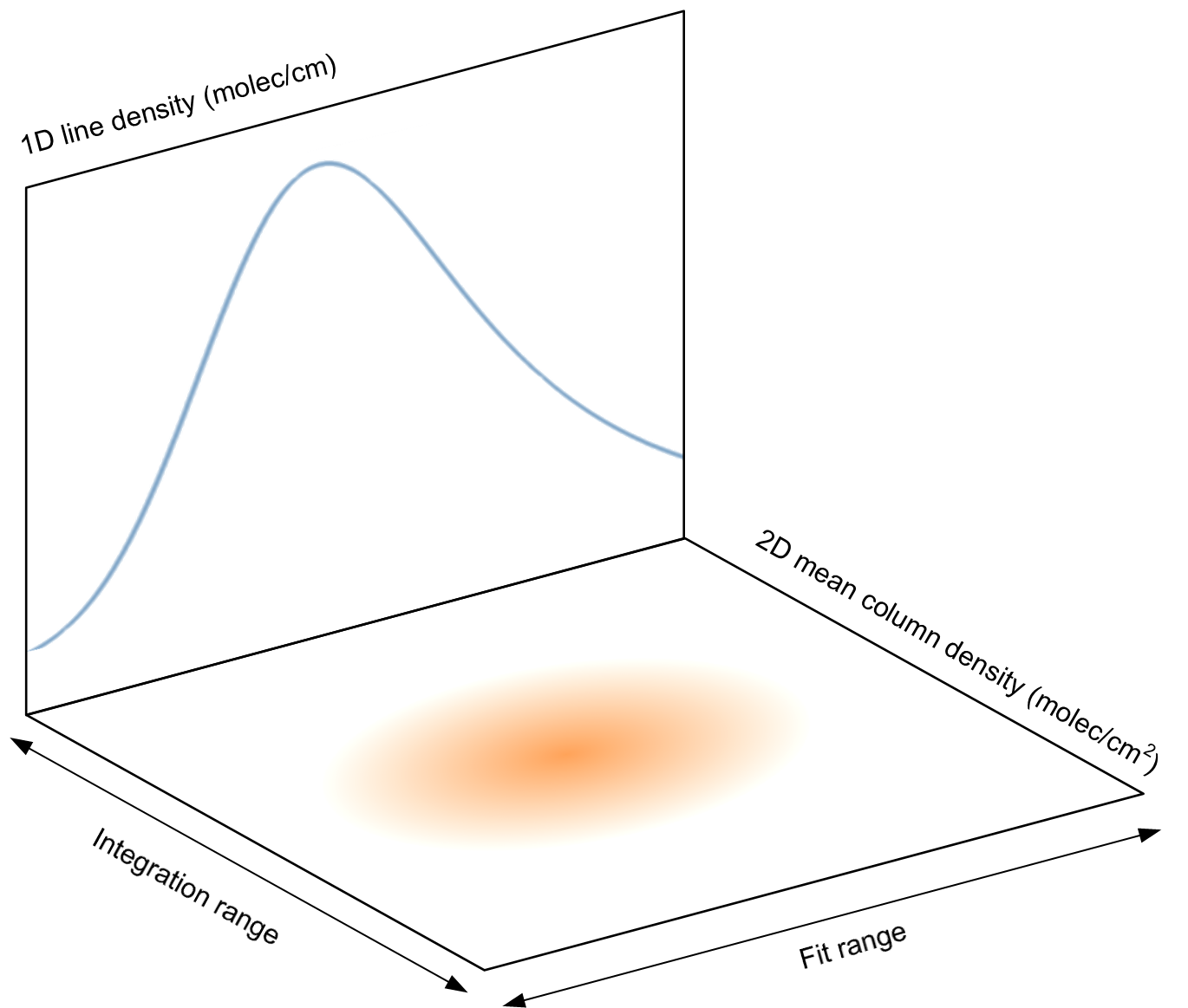


Fig. S5. Sketch of the definition of line density. Reference to Figure S1 in Beirle’s study (*10*).


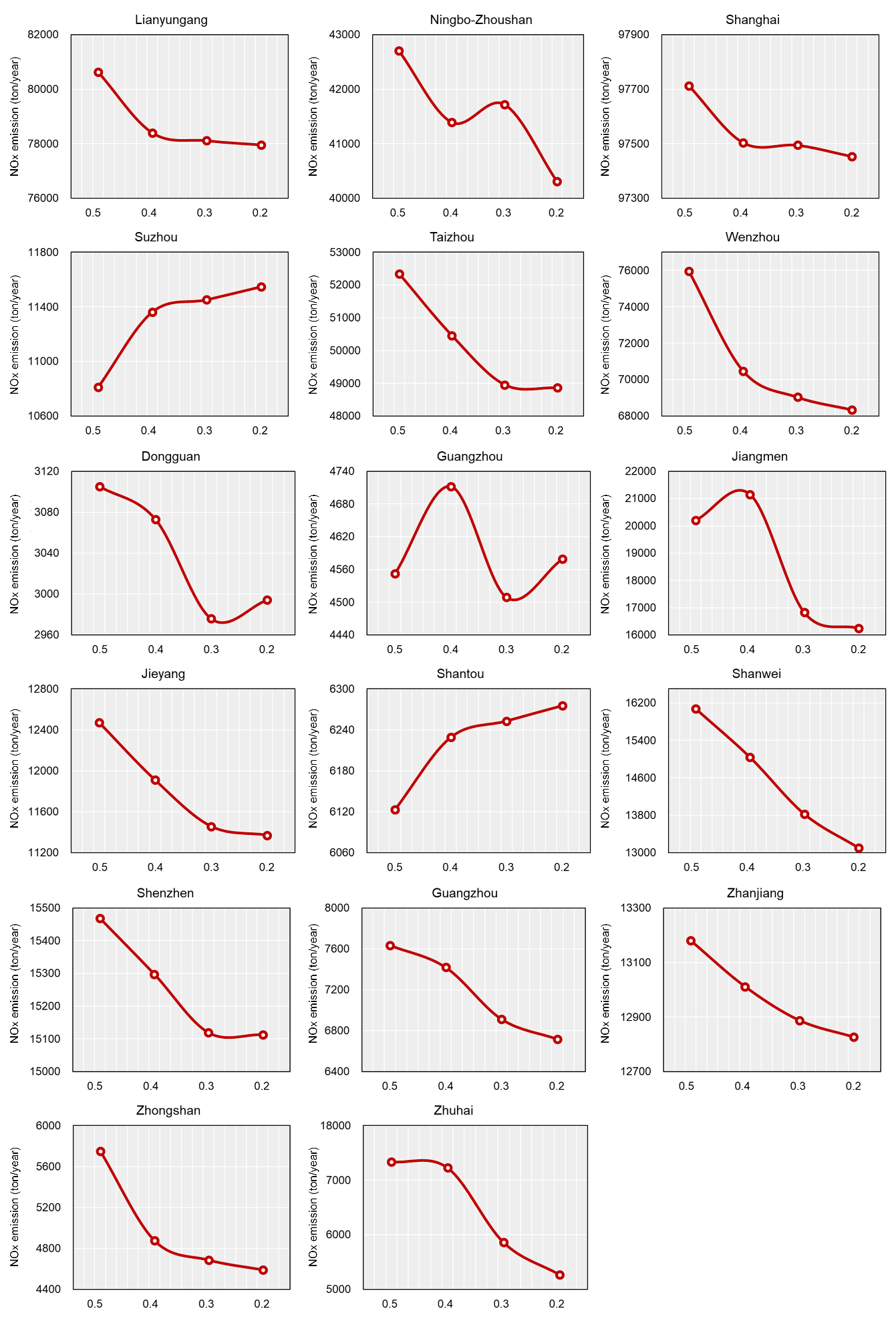


Fig. S6. The derived shipping NOx emission for each ports using SAT-SHIP in this study with different thresholds of 0.5 m/s, 0.4 m/s, 0.3 m/s and 0.2 m/s (x-axis) for no-wind pattern.


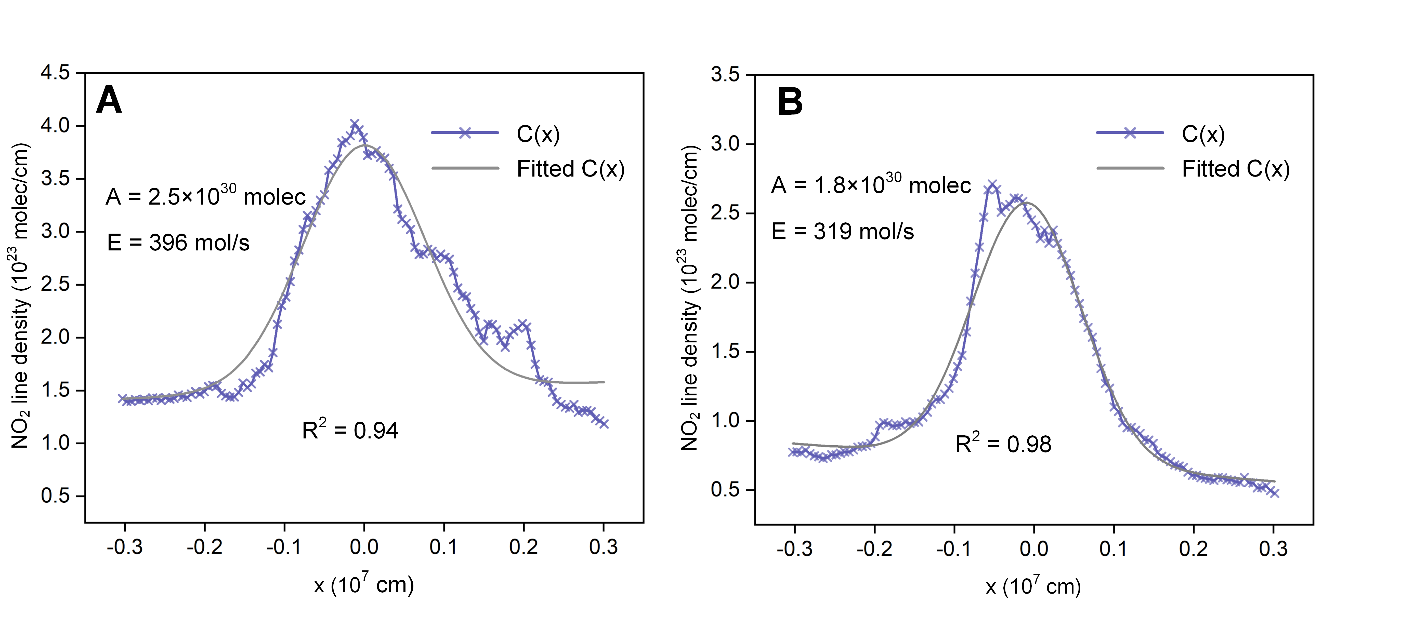


Fig. S7. The fit result $\boldsymbol{C(x)}$ for NO_2_ line densities. (A) For YRD, and (B) PRD. A is the total NO_2_ mass and E is the NOx emission.

Table S1. The shipping NOx emission for each port from SEIM and the proposed model

| Region | Ports | SEIM  (ton/year) | This study (ton/year) | | | | Standard deviation  (ton) |
| --- | --- | --- | --- | --- | --- | --- | --- |
|  |  |  | < 0.5 m/s | < 0.4 m/s | < 0.3m/s | < 0.2 m/s |  |
| YRD | Lianyungang | 12872 | 80621 | 78400 | 78117 | 77963 | 1077 |
|  | Ningbo-Zhoushan | 134021 | 142704 | 141394 | 141721 | 140310 | 855 |
|  | Shanghai | 66285 | 97712 | 97504 | 97495 | 97454 | 100 |
|  | Suzhou | 23706 | 10810 | 11363 | 11452 | 11547 | 286 |
|  | Taizhou | 33537 | 52341 | 50455 | 48955 | 48861 | 1413 |
|  | Wenzhou | 62484 | 75968 | 70473 | 69039 | 68346 | 2993 |
| PRD | Dongguan | 5504.175 | 3105 | 3073 | 2976 | 2994 | 54 |
|  | Guangzhou | 11341.97 | 4552 | 4712 | 4509 | 4579 | 76 |
|  | Jiangmen | 13680.14 | 20207 | 21154 | 16825 | 16248 | 2109 |
|  | Jieyang | 10947.31 | 12472 | 11910 | 11457 | 11371 | 438 |
|  | Shantou | 5770 | 6123 | 6229 | 6253 | 6275 | 58 |
|  | Shanwei | 9347 | 16074 | 15039 | 13828 | 13107 | 1135 |
|  | Shenzhen | 18579 | 15469 | 15298 | 15119 | 15113 | 147 |
|  | Yangjiang | 5575 | 7635 | 7419 | 6913 | 6718 | 370 |
|  | Zhanjiang | 10390 | 13181 | 13012 | 12888 | 12827 | 135 |
|  | Zhongshan | 6013 | 5751 | 4876 | 4686 | 4590 | 459 |
|  | Zhuhai | 16916 | 17340 | 17229 | 15860 | 15267 | 886 |

Table S2. The threshold for different patterns.

| Pattern | Windy | Clam | No-wind |
| --- | --- | --- | --- |
| Wind velocity | > 2 m/s | < 2 m/s | < 0.2 m/s |

**SI References**

1. M. Reichstein, G. Camps-Valls, B. Stevens, M. Jung, J. Denzler, N. Carvalhais, Prabhat, Deep learning and process understanding for data-driven Earth system science. *Nature* **566**, 195-204 (2019).

2. H. Liu, M. Fu, X. Jin, Y. Shang, D. Shindell, G. Faluvegi, C. Shindell, K. He, Health and climate impacts of ocean-going vessels in East Asia. *Nature Climate Change* **6**, 1037-1041 (2016).

3. H. Liu, Z.-H. Meng, Z.-F. Lv, X.-T. Wang, F.-Y. Deng, Y. Liu, Y.-N. Zhang, M.-S. Shi, Q. Zhang, K.-B. He, Emissions and health impacts from global shipping embodied in US–China bilateral trade. *Nature Sustainability* **2**, 1027-1033 (2019).

4. X. Wang, W. Yi, Z. Lv, F. Deng, S. Zheng, H. Xu, J. Zhao, H. Liu, K. He, Ship emissions around China under gradually promoted control policies from 2016 to 2019. *Atmos. Chem. Phys.* **21**, 13835-13853 (2021).

5. J. Zhao, Z. Lv, L. Qi, B. Zhao, F. Deng, X. Chang, X. Wang, Z. Luo, Z. Zhang, H. Xu, Q. Ying, S. Wang, K. He, H. Liu, Comprehensive Assessment for the Impacts of S/IVOC Emissions from Mobile Sources on SOA Formation in China. *Environmental Science & Technology* **56**, 16695-16706 (2022).

6. J. P. Veefkind, I. Aben, K. McMullan, H. Förster, J. de Vries, G. Otter, J. Claas, H. J. Eskes, J. F. de Haan, Q. Kleipool, M. van Weele, O. Hasekamp, R. Hoogeveen, J. Landgraf, R. Snel, P. Tol, P. Ingmann, R. Voors, B. Kruizinga, R. Vink, H. Visser, P. F. Levelt, TROPOMI on the ESA Sentinel-5 Precursor: A GMES mission for global observations of the atmospheric composition for climate, air quality and ozone layer applications. *Remote Sensing of Environment* **120**, 70-83 (2012).

7. J. van Geffen, K. F. Boersma, H. Eskes, M. Sneep, M. ter Linden, M. Zara, J. P. Veefkind, S5P TROPOMI NO2 slant column retrieval: method, stability, uncertainties and comparisons with OMI. *Atmos. Meas. Tech.* **13**, 1315-1335 (2020).

8. F. Liu, S. Beirle, Q. Zhang, S. Dörner, K. He, T. Wagner, NOx lifetimes and emissions of cities and power plants in polluted background estimated by satellite observations. *Atmos. Chem. Phys.* **16**, 5283-5298 (2016).

9. N. Wu, G. Geng, L. Yan, J. Bi, Y. Li, D. Tong, B. Zheng, Q. Zhang, Improved spatial representation of a highly resolved emission inventory in China: evidence from TROPOMI measurements. *Environmental Research Letters* **16**, 084056 (2021).

10. S. Beirle, K. F. Boersma, U. Platt, M. G. Lawrence, T. Wagner, Megacity emissions and lifetimes of nitrogen oxides probed from space. *Science* **333**, 1737-1739 (2011).

11. S. Beirle, C. Borger, S. Dörner, A. Li, Z. Hu, F. Liu, Y. Wang, T. Wagner, Pinpointing nitrogen oxide emissions from space. *Science Advances* **5**, eaax9800 (2019).
